# Practical implementation of the partial ordering continual reassessment method in a Phase I combination‐schedule dose‐finding trial

**DOI:** 10.1002/sim.9594

**Published:** 2022-11-25

**Authors:** Pavel Mozgunov, Thomas Jaki, Ioannis Gounaris, Thomas Goddemeier, Anja Victor, Marianna Grinberg

**Affiliations:** ^1^ MRC Biostatistics Unit University of Cambridge Cambridge UK; ^2^ Computational Statistics Group, University of Regensburg Regensburg Germany; ^3^ Merck Serono Ltd., an affiliate of Merck KGaA Feltham UK; ^4^ Biostatistics, Epidemiology & Medical Writing Merck Healthcare KGaA Darmstadt Germany; ^5^ Present address: Marianna Grinberg, Statistical Sciences and Innovation UCB Monheim Germany

**Keywords:** combination study, dose‐finding, dose‐schedule, partial ordering

## Abstract

There is a growing medical interest in combining several agents and optimizing their dosing schedules in a single trial in order to optimize the treatment for patients. Evaluating at doses of several drugs and their scheduling in a single Phase I trial simultaneously possess a number of statistical challenges, and specialized methods to tackle these have been proposed in the literature. However, the uptake of these methods is slow and implementation examples of such advanced methods are still sparse to date. In this work, we share our experience of proposing a model‐based partial ordering continual reassessment method (POCRM) design for three‐dimensional dose‐finding in an oncology trial. In the trial, doses of two agents and the dosing schedule of one of them can be escalated/de‐escalated. We provide a step‐by‐step summary on how the POCRM design was implemented and communicated to the trial team. We proposed an approach to specify toxicity orderings and their a‐priori probabilities, and developed a number of visualization tools to communicate the statistical properties of the design. The design evaluation included both a comprehensive simulation study and considerations of the individual trial behavior. The study is now enrolling patients. We hope that sharing our experience of the successful implementation of an advanced design in practice that went through evaluations of several health authorities will facilitate a better uptake of more efficient methods in practice.

## 1 INTRODUCTION

The primary objective of Phase I clinical trials is to study the intervention's safety and, if reached, to identify the highest safe dose of a novel agent and recommend a dose and schedule to be used in subsequent studies. The precision of the dose selection in the initial Phase I study has been shown to be decisive for the success of the complete development process.^1^ Motivated by unmet medical need in the past decades, there is growing interest in combining several agents to enhance efficacy with acceptable tolerability.^2^ Specialized approaches to how these combinations should be tested in clinical trials are required throughout the whole drug development process. A frequent objective of Phase I combination dose‐escalation clinical trials is to study the safety and tolerability of the combinations and to identify at least one (as there might be multiple—various combinations of doses might result in similar toxicity risk) maximum tolerated combination (MTC), the highest combination with an acceptable risk of toxicity. In a combination setting, in which the dosing of both agents is sought, this objective has proven to require a different approach to the identification of the maximum tolerated dose (MTD) as in monotherapy clinical trials. As a result, a number of methods to identify the MTC have been proposed in the literature in the last two decades.^3^, ^4^, ^5^ Despite a number of available alternatives and the fact that more efficient statistical methods in Phase I clinical trials can noticeably increase the probability of the success by the end of Phase III studies,^1^ their uptake in clinical practice remains low.^6^

Another emerging field in early phase drug development is dose‐schedule clinical trials in which the safety and tolerability of a monotherapy administered using various schedules is studied. These also require specialized approaches to ensure that the target dose‐schedule regimen is identified accurately.^7^ Several methods for such settings have been proposed in the literature^8^ but again these are rarely implemented in practice.

Building on the ideas of combining various treatments and administering them under different dosing schedules, there is also an interest in combination‐schedule clinical trials.^9^ Specialized advanced methods that combine the features of the designs for combination and dose‐schedule problems, and, effectively, are tackling a search of the target treatment in multiple dimensions, have been proposed recently^10^, ^11^ but, again, focused primarily on statistical aspects of designs, rather than on how these can be tailored for the need of a specific clinical trial and considered special cases with a small number of combination‐schedules.

One of the reasons for the slow uptake of more efficient clinical trial designs in early phase trials was found to be the absence of guidance in the literature on how novel methods can be implemented in actual clinical trial settings.^12^ Without this guidance, more advanced methods will remain a hypothetical statistical exercise rather than a practical way for more efficient decision‐making. This problem has recently been recognized and examples of the implementation of model‐based designs in Phase I clinical trial with a time‐to‐event outcome,^13^ a randomized Phase I clinical trial,^14^ and a seamless Phase I/II clinical trial^15^ have been published. Moreover, general guidance on the implementation of the model‐based design for single‐agent clinical trials, the continual reassessment method,^16^ R‐packages,^17^, ^18^, ^19^, ^20^ and web applications to ease the methods implementation^21^, ^22^ have been developed. However, published examples of the implementation of advanced clinical trials designs in combination or dose‐schedule clinical trials are sparse to date.^23^, ^24^

In this work, we share our recent experience of extending and implementing an advanced model‐based dose‐finding design in a three‐dimensional combination‐schedule oncology clinical trial with many possible combination‐schedules. We have extended the partial ordering continual reassessment methods (POCRMs) proposed by Wages et al^25^ and tailored it to the specific needs of the clinical trial. We focus on how a suitable clinical trial design was developed through close collaboration between the statisticians and the clinicians, how all required clinical trial design parameters were defined, how the performance of the design was evaluated and how these were communicated to the study team and health authorities. While motivated by an oncology clinical trial, we note that the proposed design and its set up also apply to dose‐finding in‐patients trials in other therapeutic areas.

## 2 MOTIVATING TRIAL

This work is based on an actual oncology clinical trial (ClinicalTrials.gov Identifier: NCT04170153) conducted by Merck KGaA and for which the authors serve as statistical and clinical collaborators. The clinical trial will study the combination of an already approved agent niraparib (denoted by N), and a novel experimental agent M1774 (denoted by M), both administered orally once daily. There are two doses of niraparib, 200 mg (approved monotherapy dose) and 100 mg. There are five doses of M1774 that can be potentially investigated, 30, 60, 90, 130, and 180 mg. In addition to the five doses of M1774, two its administration schedules, S1 and S2, were defined. Schedule S1 refers to continuous once daily dosing of M1774, whereas S2 incorporates breaks from treatment and is approximately half as intensive as S1 over the dose‐limiting toxicity (DLT) evaluation window of 28 days. Therefore, the underlying dose‐finding problem is three‐dimensional: 5 doses of M1774, 2 schedules of M1774, 2 doses of niraparib, resulting in 20 combination‐schedules (to which we will refer to as to regimens) that could potentially be tried in the trial. The primary objective is to find the regimen that corresponds to a DLT risk in the target range of (20%,35%), and recommend it for further studies. The study has been opened for enrolment, with the first cohort of patients in the study being dosed at 200 mg of niraparib, 90 mg of M1774 using schedule S1, based on prior knowledge and assumptions about the expected toxicity.

It is known prior to the trial that the toxicity of a regimen increases with the dose of each agent, and with the use of a more intensive schedule. However, the order of regimens that correspond to an increase (decrease) in doses and less (more) intensive dosing is unknown. For example, it is unknown prior to the trial whether a two‐fold increase in a single dose of M1774 for a half as intensive schedule of M1774 (with the dose of niraparib being unchanged) will result in an increase or decrease in the probability of a DLT; or whether a decrease in the dose of N while increasing the single dose of M1774 and using more intensive schedule will increase or decrease the risk of DLT. This results in a number of plausible ways of how the regimens could be ordered with respect to their monotonically increasing toxicity. We will refer to these as *orderings*, and we will call the ordering “feasible” if it satisfies the monotonicity assumption within each agent (with another agent being fixed).

As a result, a model‐based design for monotherapies that relies on the monotonicity assumption of the dose‐toxicity curve could not be applied in this setting.^26^ Furthermore, given the scheduling part of the trial, many of existing dose‐finding designs for dual‐agent combinations could not be applied as well, and a bespoke approach to the design of the trial was needed. In fact, the stated dose‐finding problem is three‐dimensional with three escalating compounds that contribute to the overall regimen's toxicity: (i) single dose of niraparib; (ii) schedule of M1774; and (iii) single dose of M1774. Given the dimensionality of the problem, it was decided to apply a flexible model‐based approach called the POCRM^25^ that can relax the monotonicity assumption in a multi‐dimensional trial.

We present a modified POCRM design below and then outline the specific arguments why it has been chosen for the considered trial.

## 3 PARTIAL ORDERING CONTINUAL REASSESSMENT METHOD

### 3.1 Methodology

Assume that R feasible orderings of the regimens satisfying the monotonicity assumption within each agent are considered. Let i be the index of the regimen, i=1,…,20, r be the index of ordering, r=1,…,R, $\pi_{ir}$ be the standardized regimen level at regimen i under the ordering r, and let $p_{ir}$ be the corresponding probability of a DLT. Then, the regimen‐toxicity model takes the form

(1) $$p_{ir}=\pi_{ir}^{\exp(\alpha_{r})},$$

where $\alpha_{r}$ is the (scalar) model parameter under the ordering r that has a normal prior distribution, $𝒩(\mu ,\sigma^{2})$, with the density function denoted by $f_{0}$. The working models $\pi_{ir}$ are constructed from standardized values, ${\tilde{\pi }}_{i}$ (also known as skeleton) by reordering them according to the order r.

As an illustration of reordering, consider a simple dual‐agent study of agents A and B, with two doses of each ($A_{1},A_{2},B_{1},B_{2}$) resulting in four combinations. There are two possible orderings of these combinations if one assumes monotonicity within each agent. Assuming the skeleton π=(0.10,0.20,0.30,0.40), the two working models are given in Table 1. This toy example was found to be an efficient tool to communicate the basic principles of the POCRM approach to the clinical team at the start of designing of the study.

**TABLE 1:** Working models consistent with each ordering

	Combinations			
Ordering	($A_{1};B_{1}$)	($A_{2};B_{1}$)	($A_{1};B_{2}$)	($A_{2};B_{2}$)
1	${\left(0.10\right)}^{\alpha_{1}}$	${\left(0.20\right)}^{\alpha_{1}}$	${\left(0.30\right)}^{\alpha_{1}}$	${\left(0.40\right)}^{\alpha_{1}}$
2	${\left(0.10\right)}^{\alpha_{2}}$	${\left(0.30\right)}^{\alpha_{2}}$	${\left(0.20\right)}^{\alpha_{2}}$	${\left(0.40\right)}^{\alpha_{2}}$

Under each ordering r, a working model is fitted using the Bayesian framework. Let $n_{j}(i)$ be the number of patients received regimen i after j cohorts have been enrolled in the study, and let $y_{j}(i)$ be the number of DLTs observed in these $n_{j}(i)$ patients. Here, the cohort is a small group of patients (typically between 1 and 5 patients). Then, under ordering $r^{\prime }$ after j cohorts have been enrolled in the trial, the likelihood is given by

(2) $$L_{r^{\prime }}(\alpha_{r^{\prime }}|n_{j}(1),\ldots ,n_{j}(20),y_{j}(1),\ldots ,y_{j}(20))=\overset{20}{\underset{i=1}{\prod }}{\left(\pi_{ir^{\prime }}^{\exp(\alpha_{r^{\prime }})}\right)}^{y_{j}(i)}{\left(1-\pi_{ir^{\prime }}^{\exp(\alpha_{r^{\prime }})}\right)}^{n_{j}(i)-y_{j}(i)}.$$

Then, the posterior distribution of $\alpha_{r^{\prime }}$ takes the form

(3) $$f_{j}(\alpha_{r^{\prime }})=\frac{L_{r^{\prime }}(\alpha_{r^{\prime }}|\cdot )f_{0}(\alpha_{r^{\prime }})}{\int_{\mathbb{R}}L_{r^{\prime }}(\alpha_{r^{\prime }}|\cdot )f_{0}(\alpha_{r^{\prime }})d\alpha_{r^{\prime }}}$$

with the mean of this distribution denoted by ${\hat{\alpha }}_{r^{\prime }}$. Then, assuming that each of r=1,…,R considered orderings had a prior probability of being the true one equal to $q_{01},\ldots ,q_{0r},\ldots ,q_{0R}$, the posterior probability of ordering $r^{\prime }$ being the correct one after j cohorts have been enrolled in the trial is given by

(4) $$q_{jr^{\prime }}=\frac{q_{0r^{\prime }}\int_{\mathbb{R}}L_{r^{\prime }}(\alpha_{r^{\prime }}|\cdot )f_{0}(\alpha_{r^{\prime }})d\alpha_{r^{\prime }}}{\sum_{r=1}^{R}q_{0r}\int_{\mathbb{R}}L_{r}(\alpha_{r}|\cdot )f_{0}(\alpha_{r})d\alpha_{r}}.$$

The above inference is performed for the working model under each ordering r=1,2,…,R, and the ordering corresponding to the maximum probability of being the correct one, $r^{⋆}$, is found. This most likely model is used to make dose‐escalation decisions for the next cohort of patients using a prespecified criterion. Using the parametric model and by using all available DLT information for the decision‐making, the model supports finding of the optimal dose of administration for the two treatments to trigger further development. We provide the proposed design based on the POCRM inference in Section 3.3.

### 3.2 Rationale for choosing POCRM

A number of advanced dose‐escalation designs for the dual‐agent combination trials within the framework of a Bayesian logistic regression model (BLRM) approach have been proposed and applied in practice over the past decade.^27^, ^28^ The distinguishing feature of these designs is that they aim to model a combination‐toxicity relationship and, hence, use more flexible parametric models compared to the POCRM. These methods allows to borrow strength from past trials (or on‐going trials) under the assumption of exchangeability.^20^, ^29^, ^30^ However, these methods typically concern the escalating doses of two agents with no consideration of changing schedules. While these methods can be applied for the dual‐agent dose‐escalation trial with various dose‐schedules, they would require an additional assumption such as that the drug exposure is fully driven by the total dose or dose intensity.

To deal with the problem of various schedules that allow relaxation of this assumption, a number of model‐based designs have been proposed recently.^31^, ^32^, ^33^ These approaches, however, concern only single agent administered under various schedules and does not cover the dual‐agent combinations.

A natural extension of these approaches would be to use a model with as many parameters as the factors contributing to the toxicity and (possibly) their interaction(s), that is, modeling schedules and total average dose through additional covariates. While in the dual‐agent combination setting, this would typically result in 4 to 5 parameters,^27^, ^34^, ^35^ in the considered trial that would result in at least 5 (4 contributing factors and intercept) and their interactions. It was found recently that it can be challenging to estimate a model with that many parameters in the setting of typical Phase I sample sizes^36^ and limited information about the agents. The latter was the case in the motivating trial as only one schedule of the M1774 was studied at the time of the planning of the trial.

The fundamental idea of the POCRM is that it benefits from the one‐parameter model within each of the considered orderings r=1,…,R but accounts for the uncertainty in the toxicity through the prespecified orderings. This flexibility allows it to be applied in both a two‐dimensional setting of dual‐agent combination, as originally proposed by Wages et al^25^ as well as the hereby considered three‐dimensional setting since it requires only orderings to define the uncertainty in their toxicity relationship. Given the number of factors that can contribute to the overall toxicity in the considered trial, the flexibility of specifying orderings coming from various assumptions (including preclinical experience, clinical data from individual combination partners, competitive data and clinical expertise) was considered a major advantage. In essence, there was no readily available alternative that could be implemented in the considered three‐dimensional dose‐finding problem in a timely manner. At the same time, the ability of the POCRM to incorporate prior clinical knowledge and assumptions on the main drivers of the toxicity through various number of orderings and their respective probabilities was considered as an advantage. We note that this approach is different to the prior knowledge incorporation in the BLRM‐type approaches that typically include the historical information through the prior on the model parameters themselves.

Furthermore, the clinician knowledge about the agents based on monotherapy experience with both niraparib and M1774 resulted in a decision for the first cohort of patients to receive a combination in the middle of the grid of regimens. The trial team expects that if a tolerable toxicity profile is observed with the starting regimen, many of the considered regimens and dose/schedule levels might not be tried in the trial at all (eg, 100 mg of niraparib or schedule S2). These de‐escalating regimens serve as backup if unexpected toxicities occurred. If certain schedules or doses will not be tested, then it would not be feasible to accurately estimate the corresponding model parameters. However, it is unknown prior to the start of the trial which doses/schedule levels will actually be tried. In other words, the modeling of the dose‐schedule combination toxicity relationship with respect to all potential drivers of toxicity was not the primary objective of the trial that the BLRM approaches are aimed at. Rather, the goal is to define a set of admissible dose‐schedule combinations that the SRC can select from for the next cohort

While this manuscript addresses the specific trial setting, more generally, early phase clinical trials with more than one (monotherapy) or two (dual‐agent combination) factors contributing to the overall toxicity become more widespread. Depending on the number of factors contributing to the overall toxicity, starting combinations and assumptions about how these factors relate to each other, each of subsequent trial may require setting up a new parametric model. Utilizing a flexible POCRM approach in this trial allows setting up an infrastructure, as a strategic resource, for application beyond this trial.

These reasons together have served as the main motivation for opting for the POCRM for the considered trial rather than alternative approaches mentioned above. We specify below how this model was used in the trial design.

### 3.3 Dose‐escalation design

The POCRM model defined above will be used to drive escalation/de‐escalation subject to several restrictions that were not originally defined in the POCRM design^25^ but were agreed to be an integral part of the considered trial. The escalation restrictions are

- Escalate only to a regimen that is considered safe. Regimen i is considered to be safe if
(5) $$\mathbb{P}\left(p_{ir^{⋆}}>0.35\right)<c_{\text{overdose}},$$
  where $c_{\text{overdose}}$ is a probability constant controlling overdosing (chosen via simulations—see details in Section 5), 0.35 is the upper bound of the target toxicity interval, and $r^{⋆}$ is the most likely correct ordering.
- When escalating, the next regimen is limited to an increase in only one dimension at time (eg, dose of M1774, dose of niraparib, or schedule);
- The total average dose per week or single dose of M1774 cannot be more than doubled compared to the current dose;
- If at least one DLT in the current cohort of patients is observed, the total average dose of M1774 or dose of niraparib cannot be increased.

The proposed design takes the following form:

1. The first cohort is allocated to the starting regimen defined by the trial team.
2. After the DLT outcomes for the previous cohort of participants are evaluated, the POCRM fits a model under each of the R orderings and finds the posterior distribution of $\alpha_{r}$ under each of them. Then, the ordering corresponding to the highest posterior probability of the ordering being the true one, $r^{⋆}$, is selected.
3. The set of admissible regimens under the most likely ordering $r^{⋆}$ satisfying the escalation restrictions above are found.
4. Among the admissible regimens, the regimen i corresponding to the minimum of
(6) $$\delta_{i}=𝔼\left(\frac{{\left(p_{ir^{⋆}}-\gamma \right)}^{2}}{p_{ir^{⋆}}^{b}{\left(1-p_{ir^{⋆}}\right)}^{\left(2-b\right)}}\right)$$
  is recommended to the next cohort of patients, where γ is the middle of the target toxicity range, $p_{ir^{⋆}}$ denotes the toxicity at regimen i under the most likely ordering $r^{⋆}$, b is an asymmetry parameter, and the expectation is taken with respect to the posterior probability $f_{j}(\alpha_{r^{⋆}})$.
5. Steps 2 to 4 are repeated until the maximum number of patients is reached or the trial is stopped for safety (all regimens are unsafe).

The criterion (6) was proposed by Mozgunov and Jaki^37^ and takes into account both the uncertainty in the estimates and allows to penalize overdosing more severely than underdosing. The asymmetry parameter b allows to tackle this balance explicitly if there are some concerns in the escalation being too aggressive (reflected, for example, in a high average number of DLTs). At the same time, it is known that for a=2×γ, the criterion (6) is nearly equivalent to the squared (or absolute) distance. Furthermore, the proposed design recommends the allocation to the regimen having an estimated toxicity risk closest to the target,

It is important to emphasize that the proposed model is serving to support the decision‐making rather than completely replace the clinical judgment (and other relevant non‐DLT information) when recommending the next regimen. Therefore, the primary role of the proposed design is to define the set of admissible regimens to which the next cohort of patients can be assigned, and to summarize and quantify the DLT risks at the prespecified regimens to the Safety Monitoring Committee (SMC). The final decision on de/escalation will be taken by the SMC and will also take into account the cause of DLT. This implies that the recommendation of the model is not binding and can be overruled by the SMC. This was explicitly mentioned in all discussions with the trial's team, SMC's members, protocol and statistical analysis plan. However, at the planning stage, it is crucial to understand how this can affect the properties of the design. We will investigate how the deviation of the recommended escalation/de‐escalation affects the operating characteristics in Section 5.

## 4 HOW TO CHOOSE ORDERINGS FOR THE POCRM

An integral part of relaxing the monotonicity assumption in the POCRM design is through prespecifying a set of orderings reflecting various assumptions on potential main drivers of toxicity in the regimens. Throughout the discussions with the clinical team, it was crucial to communicate that these orderings are the main mean to mitigate the risk of overly strong assumptions on the order of drivers of toxicity, and allow for the uncertainty in defining these drivers a‐priori. At the same time, it stresses that their choice is important. The prespecification of these toxicity orderings can have a noticeable impact on the performance of the POCRM design.^3^, ^38^ Even though Wages and Conaway^39^ proposed a framework on how to prespecify toxicity orderings for a combination setting to obtain a robust performance in a generic dual‐agent setting, each study setting might require individual adjustments to the proposed orderings to reflect compound‐specific toxicity assumptions. This is particularly relevant for the considered three‐dimensional setting in which escalation/de‐escalation decisions are made for two treatments while additionally permitting schedule changes. This was one of the most crucial part of incorporating the prior clinical knowledge in the proposed design and required the most number of discussion and interaction with the clinical team. Below, we provide the steps that were taken to define the toxicity orderings in the considered trials.

### 4.1 Specifying combination‐schedule grid

The first step of specifying the toxicity orderings is to understand all potential drivers of toxicity that can influence the toxicity outcomes in patients. The clinical team formulate that these are (i) dose of niraparib, (ii) total average dose of M1774, (iii) schedule of M1774, and (iv) the single dose of M1774. The second step was to present all possible regimens as a grid to support the visualization of all of the combination/schedules that could be tried in the trial to ensure aligned thinking between the clinical and statistical teams, and to formalize the monotonicity assumptions within each treatment and treatment schedule that are known to the trial team a‐priori. The toxicity assumptions can also incorporate any additional information on the relationships between the regimens that do not bear the uncertainty in the toxicity ordering according to the clinical judgment. It is important that all such assumptions of the certainty in the toxicity orderings are incorporated in trials relaxing monotonicity assumptions as it will results in gains in the accuracy and safety of the trial.^9^ The main challenge here was to present the three‐dimensional problem in the two‐dimensional table that could be easier communicated and to be understood by the team. The proposed resulting grid of regimens in the considered trial is given in Table 2.

**TABLE 2:** Combination‐schedule grid of all 20 regimens that can be studied in the considered trial

	S1 (16)			S1 (17)		S1 (18)		S1 (19)	S1 (20)
	N=200			N=200		N=200		N=200	N=200
	M=30			M=60		M=90		M=130	M=180
	[210]			[420]		[630]		[910]	[1260]
S2 (11)		S2 (12)	S2 (13)		S2 (14)		S2 (15)		
N=200		N=200	N=200		N=200		N=200		
M=30		M=60	M=90		M=130		M=180		
[105]		[210]	[315]		[455]		[630]		
	S1 (6)			S1 (7)		S1 (8)		S1 (9)	S1 (10)
	N=100			N=100		N=100		N=100	N=100
	M=30			M=60		M=90		M=130	M=180
	[210]			[420]		[630]		[910]	[1260]
S2 (1)		S2 (2)	S2 (3)		S2 (4)		S2 (5)		
N=100		N=100	N=100		N=100		N=100		
M=30		M=60	M=90		M=130		M=180		
[105]		[210]	[315]		[455]		[630]		

*Note*: S1 and S2 denote the schedule, the figure in square brackets corresponds to the average weekly dose of the novel agent M1774 (mg/wk), and the number in round brackets indicates the index of the combination‐schedule regimens.

In Table 2, the number in round brackets indexes the corresponding regimen. A move along the diagonal corresponds to a change of schedule, a horizontal move corresponds to a change in dose of M1774, and a vertical move corresponds to a change in the dose of niraparib. Each of the ‘moves’ from left/bottom to the right/top corresponds to an increase in the toxicity risk either because (i) at least one of the agents of the regimen is increased (while others remain the same), or (ii) the cumulative increase (decrease) changes in two or more factors is assumed to overtake a decrease (increase) in other(s)given the magnitude of the plausible toxicity effect.

The latter is designed to reflect the clinicians' knowledge of how much each of the factors (dose of niraparib, dose of M1774, schedule of M1774, and the total average dosage of M1774) contribute to the overall toxicity of the regimen. For example, comparing (14) and (18), the clinicians were confident that (18) is more toxic than (14) despite a decrease in the single dose of M1774 from 130 to 90 mg due to the increase in the total average dose of M1774 (from 455 to 630 mg) and change to a more intensive schedule. The latter two effects *together* are assumed to result in a larger increase in the toxicity than a decrease in a single dose of M1774. The same line of arguments were extrapolated for the rest of the grid.

In Table 2, all anti‐diagonal moves correspond to potentially unknown ordering between the regimens. For example, comparing (14) and (17) it is unknown which of these is more toxic as the total average amount of M1774 is slightly higher for (14), the single dose is higher for (14) but the schedule is less intensive. Similarly, comparing the regimens with the total amount of M1774 (eg, (12) vs (16) and (15) vs (18)) it is unknown a‐priori whether the increase in the individual doses of M is more (or less) prominent than the reduction in the schedule. Again, the same argument were extended to the rest of the grid.

### 4.2 Specifying the orderings

The combination‐schedule grid in Table 2 served as a basis to specify a number of plausible orderings to be considered in the trial. Note that there is a large number of possible orderings in the considered setting due to a large number of regimens. However, it was shown by Wages and Conaway^39^ that it is not required to specify them all for an accurate performance of the POCRM design. However, the selected ordering should cover a range of plausible orderings that can occur in the trial. It is important that even orderings that are thought to be less probable then others (but still plausible) are incorporated into the final set of orderings to relax the monotonicity assumptions and safeguard from fail to identify the right regimen in such scenarios. This was a crucial moment for the specification of the orderings as this requires the complete understanding (and explicit acknowledging) the uncertainty about the drivers of the toxicity and inclusion of those plausible scenarios that the clinician believe are less likely but still possible. The statistical team has specifically emphasized that the likelihood of each ordering is secondary at this point of the design specification, and how probable the direction of the effect between such regimen is will be defined via a‐priori toxicity probabilities further.

Following the discussion with clinicians, the assumption on the order of effect of each of the four factors, and following the guidance on the ordering specification by Wages and Conaway,^39^ a list of various orderings were provided to the team. These orderings were specified under the various assumptions of the main drivers of the toxicity. At the first iteration of the ordering discussion, the orderings were formulated as a sequence of the regimen indices, for example,

(1),(2),(3),(6),(4),(5),(7),(8),(9),(10),(11),(12),(13),(16),(14),(15),(17),(18),(19),(20)

as typically presented in the statistical literature on the POCRM. This, however, was quickly found to be a cumbersome approach. To tackle this, we have supplied each of the specified orderings with the assumption made to construct this ordering in terms of the specified driver of the toxicity. For example, some of assumptions to construct the orderings were

- Dose of niraparib has the highest effect, followed by the schedule of M1774 (for low to moderate difference in the total average amount of M1774), then the total average amount of M1774.
- Dose of niraparib has the highest effect, followed by the total average amount, followed by the schedule of M1774.
- Dose of niraparib has the highest effect up to some total average dose of M1774, followed by schedule of M1774 (for low to moderate differences in the total average dose), followed by the total average amount of M1774.

**FIGURE 1:**
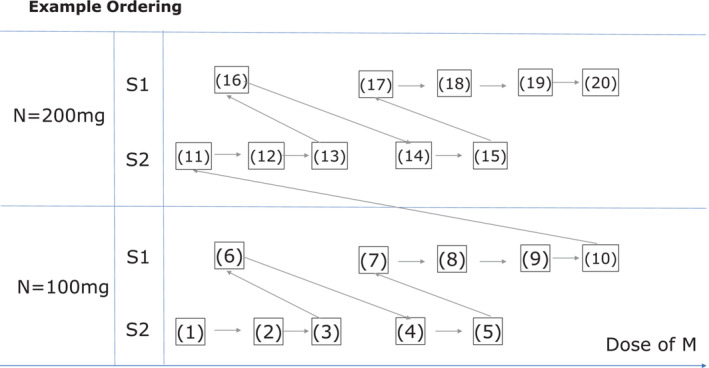
Illustration of the example ordering provided for the clinical team. The index notations correspond to the notation in Table 2 and arrows correspond to the direction of an increase in the overall toxicity

Additionally, to communicate different orderings according to various toxicity assumptions between regimens, each of them was presented as a figure displaying the increasing risk of toxicity under the made assumptions. An example of such visualization of the toxicity ordering is illustrated in Figure 1. For simplicity, the figures used the index notation from the combination‐schedule grid in Table 1. The example ordering is constructed under the assumption of dose of N having the highest effect, followed by schedule of M1774 (for low to moderate difference in the total average amount of M1774), then the total average amount of M1774.

Within each of the assumptions, following the suggestion by Wages and Conaway,^39^ we have altered the order in which the regimen would occur (eg, down diagonals, up diagonals, the mix of those). This ensured that the toxicity orderings provided to the clinicians have an intuitive interpretation, are aligned with the knowledge of the mechanism of action for the studied agents but also will later result in good operating characteristics. We have also added six orderings recommended by Wages and Conaway^39^ to achieve a robust performance of the POCRM design for the considerations by the team.

In the first iteration, fourteen orderings were provided to the clinicians, of which 10 were agreed as being plausible for the considered trial. For example, one of the orderings excluded from the consideration by the team is one of the “default” orderings proposed by Wages and Conaway^39^ referred to, originally, as “by columns”

(1),(11),(6),(16),(2),(12),(3),(13),(4),(14),(5),(15),(6),(16),(7),(17),(8),(18),(9),(19),(10),(20).

This is because of the knowledge that the backbone agent, niraparib, is considerably more toxic than the novel agent M1774 (at least for lower doses tried in the trial) and hence such an ordering will not be plausible. The set of the 10 orderings that were considered in the trial are given in the Supplementary Materials.

### 4.3 Specifying prior probabilities of each orderings

After the orderings were agreed, the POCRM requires to reflect a‐priori toxicity assumptions on how likely each of the prespecified orderings to occur during the conduct of the study. The default specification of the POCRM considered in the literature focuses on the equal a‐priori probability. However, given the clinicians knowledge about the two agents, it was agreed that not all of the considered orderings are equally likely and this should be reflected in the model specification.

The specification of the POCRM requires to assign the probability to each of the ordering directly. However, eliciting the prior probability for each of the 10 orderings can be challenging given that each of the ordering consists of 20 regimens and some of them might differ only partly and it can be difficult to grasp all of the differences between various orderings,^40^ and hence accurately elicit the prior clinical knowledge.

To tackle this, we have developed an approach of eliciting the probability ordering through the prior probabilities for pairs of anti‐diagonal regimens with uncertainty in the toxicity ordering. For example, regimen (12) and (16) that has the same total average dose of M and the same dose of N but less intensive schedule yet higher single dose of M1774. The pairs of regimens for which the prior probability of ordering was elicited and the prior probabilities provided by the clinicians are given in Table 3.

**TABLE 3:** A‐priori probabilities of order between the pairs of anti‐diagonal combinations

Pair	Elicited prior probability	Used prior probability
(12) > (16)	10%	12%
(16) > (12)	90%	88%
(17) > (14)	50%	50%
(14) > (17)	50%	50%
(18) > (15)	80%	80%
(15) > (18)	20%	20%
(13) > (16)	65%	61%
(16) > (13)	35%	39%

For example, considering (12) and (16), it is believed that there is 90% probability that (16) is more toxic given that schedule is thought to have a larger effect on toxicity than the single dose of M. In contrast, comparing, (14) and (17), it is thought that there is an equal chance of one of these being more toxic than the other given that the total average dose of M1774 is slightly higher for (14) together with the single dose of M1774 but the schedule is less intensive. Similar prior probabilities were assumed to hold for 100 mg of niraparib, for example, regimen (2), (6) and (4), (7). Additionally, the clinical team argued that orderings 9 to 10 should have lower a‐priori probabilities compared to orderings 1 to 8, and the sum of the first 5 orderings (corresponding to the ordering where agent N is the dominant factor in toxicity) should be between 67% and 75% as they were quite confident that agent niraparib will be the main driver in toxicity. Note that the remaining probability of 25% to 33% safeguards from relying overly strongly on this a‐priori toxicity assumptions and enhances the identification of such orderings even if these are thought to be less unlikely.

Given these constraints, the prior probability of each complete ordering implying these prior probabilities on ordering on pair of regimens was found by grid search. The prior probabilities of ordering resulting in the lowest sum of squares of the elicited and computed prior probabilities were found and are provided in the Supplementary Materials. The prior probabilities on the pairs of anti‐diagonal regimens implied by these orderings' probabilities are given in the last column of Table 3. These prior probabilities of orderings were subsequently used in the evaluation of the proposed design.

## 5 PARAMETERS OF THE POCRM DESIGN

In this section, we finalize the design parameters required for the considered trial and evaluate the performance of the proposed design in individual escalation paths.

### 5.1 Trial parameters

The proposed POCRM design requires a number of parameters to be specified additional to the prespecified orderings. Some of these parameters are chosen through discussions with clinicians and others through statistical considerations and tuning via simulations. We consider the parameter that were specified from discussions with the clinicians below and the parameter obtained via tuning in Section 5.2.

The target toxicity range was specified as 20% to 35%, and the target toxicity level lying in the middle of this interval γ=0.275 was used for the criterion 6. The proposed criterion also requires specification of the so‐called asymmetry parameter that balances the accuracy‐safety trade‐off. As b=2γ approximately results in the standard squared distance criterion, a slightly higher value of b=0.60 (but still below the double of the upper toxicity bound 2×0.35=0.70) was used to explore the combination‐schedule grid more thoroughly as higher values of b were found to result in higher accuracy.^37^ The cohort size of c=3 patients is used, and the sample size is to be defined by simulations but maximum sample sizes between 42 and 54 are to be considered. The trial will start at the regimen (18).

### 5.2 Selecting the design parameters

The proposed design requires specification of several model‐specific parameters including the skeleton ${\tilde{\pi }}_{i}$, prior mean μ, prior variance $\sigma^{2}$, and the overdosing threshold $c_{\text{overdose}}$. After the prior clinical knowledge about the agents was included via the specified probabilities of each ordering, these were selected via the grid search by conducting extensive simulations for various combinations of values of these parameters and searching for the one yielding high accuracy.^36^ Note that the skeleton requires 20 values to be specified. To ease the procedure, they were defined in terms of the spacing, ν, between the neighboring combinations ${\tilde{\pi }}_{i}=0.01+\nu \times i$ where i=0,…,17 and the last two values (the escalating part of the grid—regimens (19) and (20)) were tuned separately from the escalation conditions below. The following grids were used: μ={0,0.50,0.55,0.80,1,1.25,1.40,0.15,1.75}, $\sigma^{2}=\{0.40,0.45,0.50,0.75,1.0,1.25,1.34,2.0\}$, $c_{\text{overdose}}=\{0.25,0.30,0.50\}$, ν={0.025,0.03,0.035,0.04,0.0425}.

While this was agreed that the design parameters could be chosen from the statistical considerations, that is, from the accuracy and safety in the simulation study, it was essential that the resulting parameters yield intuitive dose‐escalation/de‐escalation decisions for the first three cohorts of patients. Specifically, using the search over the grid specified above, any combination of values within this grid is excluded if they do not lead to the following escalation/de‐escalation decisions for the first cohort of 3 patients. Specifically, after 0 DLTs, it was required that (19) is the next safe regimen and (20) is yet inadmissible, and after 1 DLT on the starting regimen, regimens (14) and (17) are in the admissible set while the starting regimen (18) is not. This additional step was taken to overcome the “black‐box” concern. Then, for these combinations of parameter value, a simulation study across various scenarios was conducted using 1000 simulations for each scenario. Then, the design parameters resulting in the highest geometric mean in the proportion of correct regimen selection is taken forward for further evaluation. The list of the design parameters tried are given in Table 4.

**TABLE 4:** Set of values (options) of parameters studied in calibration with the set of values marked by star chosen to be studied further

Option	(1)	(2)	(3)	(4)${}^{⋆}$	(5)	(6)	(7)	(8)	(9)	(10)
Prior mean, μ	0.80	1.00	1.25	1.50	1.75	1.25	1.40	0.50	0.55	0.00
Prior variance, $\sigma^{2}$	0.45	0.50	0.75	1.00	1.25	1.00	1.34	1.34	2.00	0.40
Overdose prob $c_{\text{overdose}}$	0.25	0.25	0.25	0.25	0.25	0.30	0.30	0.50	0.50	0.50
Skeleton spacing, ν	0.03	0.035	0.035	0.04	0.0425	0.04	0.04	0.035	0.04	0.025

To choose scenarios for calibration, one can opt for a limited number (around four) of qualitatively different scenarios with the optimal regimens being located in various parts of the grid.^36^ However, due to a low computational costs of the one‐parameter working model, we have performed the grid search using 20 scenarios that were agreed to be clinically plausible in the considered trials and were subsequently used for the design's evaluation (see Section 6.2 for further details on scenarios).

The set (4) in Table 4 was found to result in a high proportion of the correct selections and controlling overly toxic selections. Hence, the prior mean μ=1.50, prior variance, $\sigma^{2}=1.50$, overdose threshold $c_{\text{overdose}}=0.25$, and the skeleton values

(0.01,0.05,0.09,0.13,0.17,0.21,0.25,0.29,0.33,0.37,0.41,0.45,0.49,0.53,0.57,0.61,0.65,0.69,0.75,0.80)

were chosen for the POCRM design.

### 5.3 Individual trial behavior

An important aspect of a model‐based design is that its escalation and de‐escalation recommendations are aligned with the clinician's knowledge and expectations. To communicate the model's recommendations, Yap et al^41^ developed dose‐transition pathways (DTP) as a tool that displays a decision‐tree of the models recommendation were a particular number of DLTs have been observed in previous cohorts.

**FIGURE 2:**
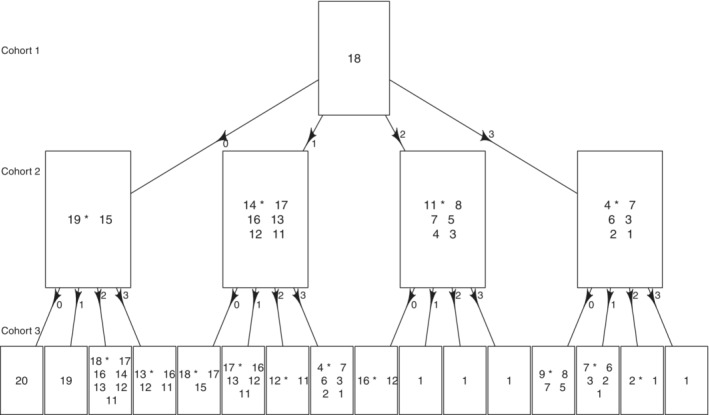
Example of the dose‐transition pathways including the set of admissible regimens recommended by the model for the first 3 cohorts (each with 3 participants) depending on the number of experienced DLTs (from 0 to 3) and using minimization of the normalized distance criterion

While the original DTP and its implementation^42^ focuses on the one‐parameter CRM model for single‐agent trials, we have extended the DTP to allow for several candidate regimens (fulfilling the admissibility criteria in Section 3.3) to be recommended by the model. An example of the decision‐tree provided to the team using the selected values of the design parameters is given in Figure 2.

Importantly, the first version of the decision‐tree included only one regimen per box as in the original DTP proposal, that is, the regimen recommended by the model according to the criterion (6) and subject to the safety constraint. However, the primary use of the proposed model is not only to recommend the single regimen for the next cohort but to define the set of admissible regimens that the SRC can select from for the next cohort. Therefore, such a tree was agreed not to convey the required message. Additionally, to the recommended regimen (marked with a ⋆), the later version of the tree has also included the admissible set to illustrate the choice the SRC will have at the given step of the trial. At the same time, as within the given path, there might be several admissible regimens, plotting a tree for each case will make the decision‐tree infeasible to read and communicate. Therefore, within each path, the regimen recommended using the model's criterion 6 drives the trajectory of the remaining paths. This amendment, however, led to another challenge of fitting all the admissible regimens within the same decision‐tree, as there could be many. For example, if no DLTs are observed, all regimens will be deemed admissible. To facilitate clearer communication and as the clinicians are primarily interested in the approved 200 mg of niraparib, we have applied additional constraints to make the tree easier to communicate. Specifically, if there are multiple admissible regimens with 200 mg of niraparib, only these are displayed. If there is only a single regimen like this, then it is displayed together with regimens corresponding to 100 mg of niraparib and to no more than halving of the total average dose of M. Otherwise, all admissible regimens are displayed.

The decision‐tree starts from the regimen (18). If no DLT is observed in the first cohort of 3 patients, the set of admissible regimens is (15) and (19) that correspond to an increase in the dose of M. Among these two, the regimen (19) would be recommended by the model. Note that, as required, the regimen (20) is not in the admissible set. We see that it is only possible to escalate to regimen (20) if no DLTs are also observed for the second cohort of patients.

If 1 DLT is observed for the first cohort, the regimens (14) and (17) are in the admissible set of regimens together with lower regimens, and the model recommends de‐escalating to regimen (14). If no DLT is observed at this regimen, the model will recommend to escalate back to the starting regimen (18).

If 2 DLTs is observed in the first cohort, there is only one regimen with 200 mg of the agent niraparib which is safe to administer. Finally, if all 3 patients in the first cohort experience the DLT, there are no regimens with 200 mg of niraparib that are deemed safe and it is recommended to de‐escalate the total average dose of M1774.

This tree was presented to the clinical team and it was agreed to satisfy the required conditions for the first cohort and lead to intuitive escalation/de‐escalation decisions for the subsequent cohorts.

Additionally to the decision‐tree that presents only the recommended and admissible set of regimens, an example of the output with the characteristics of all 20 regimens was presented for some of the branches of the tree. This included (i) a table (with the numerical characteristics of the regimens) and (ii) a figure illustrating the probabilities of overdosing, underdosing, and being in the target range. These served as one more check that the model's recommendations are aligned with the clinicians beliefs and knowledge. This, again, would resolve the “black box” concerns about the properties of the proposed model. Additionally, this also served as a tool to initiate the process of refining the details that should be included in the actual analysis during running of the trial. We refer the reader to the Supplementary Materials for the example output and the description of characteristics of the regimens that were provided.

## 6 SIMULATION STUDY

Once it was agreed that the proposed design results in intuitive escalation/de‐escalation decisions for the first cohorts in the trial, the proposed design was extensively evaluated via a simulation study to ensure that it results has good operating characteristics in a variety of possible scenarios throughout the whole trial.

### 6.1 Simulation setting

The simulation study has used the setting of the considered trial targeting the toxicity interval of (20%, 35%) with the cohort size of 3 patients, and using the calibrated design parameters from above. Three maximum sample sizes, n=42, 48, 54, were investigated to make the decision on the sample size (the results of n=42, 54 are provided in the Supplementary Materials).

As mentioned above, based on the observed data, the model recommends a subset of admissible regimens with acceptable toxicity and provides a non‐binding suggestion for the next regimen for the next cohort from the estimated set of acceptable regimens. However, the clinical team may choose a different regimen than suggested by the model. This, however, will clearly affect the operating characteristics. Therefore, we consider two options of the allocation in our simulations.

The design under the first considered allocation criterion strictly follows criterion (6). We refer to this version as “next best.” The design under the second allocation criterion is when the allocation within the admissible set is made (which can be different from the recommended by criterion (6)). To mimic the nature of the actual trials in which more than binary DLT/non‐DLT might influence the clinicians' decisions, the allocation is implemented via randomization of the next cohort of patients within the admissible set of regimens with the probabilities proportional to the inverse of the criterion, $1/\delta_{i}$. This mimics that, on average, the regimens with the target toxicity closer to the target of 27.5% are more likely to be allocated (but is not guaranteed to be selected in the light of other considerations, eg, attributions of the observed toxicity). We will refer to it as the “within admissible set.”

We evaluate the proposed design in terms of

- Probability of *optimal* regimen selection—the regimen corresponding to the toxicity risk closest to the middle of the target interval, that is, 27.5%.
- Probability of *correct* regimen selection corresponding to 20% to 35% DLT risk.
- Probability of overly toxic (>35% DLT risk) regimen selection.
- Average number of DLTs observed in the trial.
- The probability to stop the trial for safety.
- Average sample size.

We would also explore the average performance of the design together with scenario‐by‐scenario performance. We will focus on the

1. Percentage of scenarios with the proportion of the optimal selections above 40%.
2. Percentage of scenarios with the proportion of the correct selections above 60%.
3. Percentage of scenarios with the proportion of the overly toxic selections below 25%.

These probabilities are denoted by ℙ. We explore these metrics in a number of scenarios that are defined below.

### 6.2 Scenarios

Twenty regimen‐toxicity simulation scenarios detailed in Table 5 are constructed to cover a wide range of clinically plausible cases of where the MTC can be located and various cases of the interaction mechanisms between the agents and the effect of dosing schedules.

**TABLE 5:** Regimen‐toxicity scenarios considered in the simulation study

Scenario 1										Scenario 2.1									
	0.05			0.10		0.15		0.20	**0.30**		0.10			0.15		0.20		**0.30**	0.50
0.02		0.03	0.05		0.10		0.15			0.03		0.05	0.10		0.15		0.20		
	0.02			0.05		0.10		0.15	0.20		0.03			0.10		0.15		0.20	**0.30**
0.01		0.02	0.03		0.05		0.10			0.02		0.03	0.05		0.10		0.15		
Scenario 2.2										Scenario 2.3									
	0.10			0.15		0.20		**0.30**	0.50		0.10			0.13		0.15		0.45	0.50
0.03		0.05	0.10		0.15		0.20			0.03		0.05	0.10		0.13		0.20		
	0.03			0.05		0.10		0.15	0.45		0.03			0.10		0.13		0.20	**0.30**
0.01		0.02	0.03		0.05		0.10			0.01		0.03	0.05		0.10		0.15		
Scenario 3.1										Scenario 3.2									
	0.15			0.20		**0.30**		0.45	0.55		0.15			0.20		**0.30**		0.45	0.55
0.05		0.10	0.15		0.20		**0.30**			0.05		0.10	0.15		0.20		**0.30**		
	0.05			0.15		0.20		**0.30**	0.45		0.05			0.10		0.15		0.20	0.45
0.03		0.05	0.10		0.15		0.20			0.03		0.05	0.08		0.10		0.15		
Scenario 3.3										Scenario 4.1									
	0.15			0.20		**0.30**		0.45	0.55		0.15			**0.30**		0.45		0.50	0.55
0.05		0.08	0.10		0.15		0.20			0.13		0.15	0.20		**0.30**		0.45		
	0.05			0.10		0.15		0.20	0.45		0.13			0.20		**0.30**		0.45	0.50
0.03		0.05	0.08		0.10		0.15			0.10		0.13	0.15		0.20		**0.30**		
Scenario 4.2										Scenario 4.3									
	0.20			**0.30**		0.45		0.50	0.55		0.20			**0.30**		0.45		0.50	0.55
0.10		0.15	0.20		**0.30**		0.45			0.10		0.13	0.15		0.20		0.45		
	0.10			0.15		0.20		0.45	0.50		0.10			0.15		0.20		0.45	0.50
0.08		0.10	0.13		0.15		0.20			0.08		0.10	0.13		0.15		0.20		
Scenario 4.4										Scenario 4.5									
	0.15			0.20		0.45		0.50	0.55		0.20			0.45		0.50		0.55	0.60
0.10		0.15	0.20		**0.30**		0.45			0.10		0.13	0.15		**0.30**		0.45		
	0.10			0.15		0.20		0.45	0.50		0.10			0.15		0.20		0.45	0.55
0.08		0.10	0.13		0.15		0.20			0.08		0.10	0.13		0.13		0.15		
Scenario 5.1										Scenario 5.2									
	**0.30**			0.45		0.50		0.55	0.60		**0.30**			0.45		0.50		0.55	0.60
0.15		0.20	**0.30**		0.45		0.50			0.15		0.20	**0.30**		0.45		0.50		
	0.15			**0.30**		0.45		0.50	0.55		0.10			0.20		0.45		0.50	0.55
0.13		0.15	0.20		**0.30**		0.45			0.08		0.10	0.15		0.20		0.45		
Scenario 5.3										Scenario 5.4									
	**0.30**			0.45		0.50		0.55	0.60		0.20			0.45		0.50		0.55	0.60
0.10		0.15	0.20		**0.30**		0.45			0.15		0.20	**0.30**		0.45		0.50		
	0.10			0.16		0.18		0.20	0.22		0.10			0.13		0.20		**0.30**	0.45
0.08		0.10	0.15		0.16		0.18			0.08		0.10	0.15		0.16		0.17		
Scenario 6.1										Scenario 6.2									
	**0.30**			0.45		0.50		0.60	0.65		**0.30**			0.45		0.50		0.60	0.65
0.20		**0.30**	0.43		0.45		0.55			0.13		0.15	0.20		0.45		0.55		
	0.15			0.20		0.45		0.55	0.60		0.13			0.20		0.45		0.55	0.60
0.10		0.13	0.15		0.20		0.45			0.10		0.13	0.15		0.20		0.45		
Scenario 7										Scenario 8									
	0.45			0.55		0.60		0.65	0.70		0.50			0.60		0.65		0.70	0.75
**0.30**		0.45	0.50		0.55		0.60			0.45		0.50	0.55		0.60		0.65		
	**0.30**			0.48		0.55		0.60	0.65		0.45			0.55		0.60		0.65	0.70
0.20		**0.30**	0.45		0.50		0.55			**0.30**		0.45	0.50		0.55		0.60		

*Note*: The optimal combinations having the toxicity risk closest to the middle of the target interval, 27.5%, are in bold print.

Scenarios are constructed by moving the anti‐diagonal at which the optimal regimen(s) is(are) located and the first figure in the scenario number corresponds to the diagonal the optimal regimen(s) is (are). For example, under scenario 1, the optimal regimen is the highest regimen. Under scenarios 2, the optimal regimen(s) is on the second to the right anti‐diagonal. Specifically, under scenario 2.1 there are two target regimens—(19) and (15), under scenarios 2.2—only regimen (19) is the target, and under scenario 2.3—only regimen (15) is the target. Following the same structure, other scenarios were constructed by moving the diagonal with the optimal doses across the grid up to scenario 8, in which the optimal dose is the lowest regimen. While the scenarios were initially constructed by the statisticians, they were then reviewed by the clinical team to ensure that they indeed reflect possible regimen‐toxicity relationships.

### 6.3 Competing approaches

We will compare the performance of the proposed POCRM design to two competing approaches. The first one is a conventional rule‐based 3+3 design (with de‐escalation). Although, there is a long record of studies showing the inferiority of the 3+3 design compared to more statistically sound approaches,^43^, ^44^, ^45^, ^46^ the 3+3 design remains the prevalent Phase I dose‐finding design and, according to our experience, a “default” option for many industry and academic investigators and for some health authorities. Specifically, we have found the direct comparison of a model‐based design to the 3+3 design in the considered clinical setting to be an effective tool in providing the trial team with the arguments in favor of the more statistical approach.

The proposed study is a dual‐agent combination trial while the 3+3 design is a method for single‐agent studies. To apply 3+3 design to the proposed trial, one needs to assume a single monotonic dose‐toxicity ordering to mimic the setting of a single‐agent trial. In the comparison below, we assume the monotonic ordering with respect to the combination numbers.

Furthermore, for the 3+3 design, we will limit consideration of combinations to 9 combination/schedules: 11, 12, …, 20 due to computational purposes. The operating characteristics of the 3+3 design are found analytically (rather than by simulations) by computing all possible paths the trial can take. With more than 9 combinations, this computation can become infeasible. Note that this assumption favors the 3+3 design in the comparison as it considers fewer combinations in the trial than the proposed design. These nine combinations are selected such that they correspond to the 200 mg of the N compound that is a more clinically interesting (and the approved) dosage of the agent. The 3+3 design is implemented using the bcrm‐package.^18^

The second competing approach, as suggested by a reviewer, is a variant of the proposed POCRM that instead of choosing one, the most likely model as originally proposed by Wages et al,^25^ uses the model averaging taking into account the probability of all orderings. Specifically, when a model under each ordering is fitted, the estimates of the quantities of interest (toxicity risk, probability of overdosing, the escalation criterion, etc.) are computed. Then, to obtain the overall estimates, these are weighted with the probability of the corresponding ordering to be the correct one. The rest of the design set up (escalation constraints, overdosing constant, escalation criterion) remain the same. The idea is that instead of choosing one ordering (that might have just slightly higher probability of being the true one than others) take into account how likely the orderings are. We use the same parameters as for the proposed POCRM for this competing approach.

It worth noting that this approach was not originally chosen for the motivating trial mainly for two reasons. First, it is more computationally expensive to fine‐tune and evaluate in a timely manner (that was an important consideration at the time of the planning of the study): given the 10 orderings, the computational costs are 10‐fold higher for this design. Second, while under the proposed approach one might select an ordering that is slightly more likely than others, the main objective of the design is to provide the SRC with the set of admissible combinations rather than a single recommendation. Hence, it was thought that the effect of choosing different model will have a modest effect in practice.

### 6.4 Numerical results

The summary of the performance of the proposed design in the 20 considered scenarios based on 2000 simulations under the sample size n=48 is given in Table 6 (with the results for n=42, 54 being reported in the Supplementary Materials).

**TABLE 6:** Operating characteristics for the proposed POCRM design, and the POCRM following (i) the allocation according to the criterion (next best), (ii) the randomization “within admissible set,” and the 3+3 design

	1	2.1	2.2	2.3	3.1	3.2	3.3	4.1	4.2	4.3	4.4	4.5	5.1	5.2	5.3	5.4	6.1	6.2	7	8	M	ℙ
*Proposed POCRM design—Next best*																						
Optimal	48	46	43	8	61	52	33	60	36	17	20	23	56	44	40	29	35	27	47	44	39	55
Correct	86	91	85	45	87	80	71	75	73	62	68	48	72	69	68	60	62	57	59	44	68	75
Overtoxic	0	4	8	33	10	13	19	18	24	32	27	31	23	26	13	27	35	32	37	27	21	50
% of DLTs	21	23	23	24	26	26	25	29	28	28	28	28	31	31	28	29	32	30	37	42	28	‐
Early stop	0	0	0	0	0	0	0	0	0	0	0	0	0	0	0	0	0	0	4	29	‐	‐
Av SS	48	48	48	48	48	48	48	48	48	48	48	48	48	48	48	48	48	48	47	41	‐	‐
*POCRM with model averaging—Next best*																						
Optimal	47	42	37	4	47	38	39	46	33	30	16	16	60	36	22	40	20	6	48	47	34	40
Correct	82	87	80	12	84	80	78	85	74	66	65	44	86	80	65	72	72	72	65	47	70	90
Overtoxic	0	3	7	26	9	13	12	8	15	17	23	31	9	12	16	16	22	20	31	22	15	85
% of DLTs	22	24	25	24	27	27	26	29	29	28	28	29	32	30	26	30	31	29	39	44	29	‐
Early stop	0	0	0	0	0	0	0	0	0	0	0	0	0	0	0	0	0	0	4	31	‐	‐
Av SS	48	48	48	48	48	48	48	48	48	48	48	48	48	48	48	48	48	48	47	41	‐	‐
*Proposed POCRM design—Within admissible set*																						
Optimal	42	39	34	11	53	49	32	56	35	17	23	21	57	41	34	30	35	20	46	43	36	40
Correct	84	89	80	49	86	78	75	76	74	64	69	45	74	69	62	67	63	57	60	43	68	80
Overtoxic	0	2	9	19	8	10	12	12	19	24	21	28	18	25	14	20	35	29	36	27	18	75
% of DLTs	21	23	23	24	26	25	24	28	27	27	27	27	32	31	27	28	32	30	38	44	28	‐
Early stop	0	0	0	0	0	0	0	0	0	0	0	0	0	0	0	0	0	0	4	30	‐	‐
Av SS	48	48	48	48	48	48	48	48	48	48	48	48	48	48	48	48	48	48	47	41	‐	‐
*POCRM with model averaging—Within admissible set*																						
Optimal	33	30	28	5	39	37	30	44	37	25	22	17	57	42	24	39	23	15	44	46	32	25
Correct	72	80	77	16	82	80	67	83	75	61	66	31	83	79	61	67	66	66	64	46	66	90
Overtoxic	0	2	6	18	5	6	6	5	9	7	17	15	11	13	8	14	25	17	31	21	11	90
% of DLTs	18	20	20	20	22	22	21	24	24	23	22	24	28	27	25	26	28	26	35	42	25	‐
Early stop	0	0	0	0	0	0	0	0	0	0	0	0	0	0	0	0	0	0	5	34	‐	‐
Av SS	48	48	48	48	48	48	48	48	48	48	48	48	48	48	48	48	48	48	46	40	‐	‐
*3+3 design*																						
Optimal	29	25	25	0	39	39	35	24	25	22	1	5	44	44	36	4	42	37	0	0	24	15
Correct	55	63	63	0	65	65	65	25	70	69	3	53	50	50	54	55	42	47	0	0	47	35
Overtoxic	0	6	6	14	9	9	9	27	29	29	22	41	48	48	43	44	52	46	74	60	31	45
% of DLTs	19	26	26	27	30	30	30	36	36	36	33	49	43	43	42	41	44	43	54	58	37	‐
Av SS	12	12	12	11	12	12	12	13	13	13	12	14	15	15	15	14	16	15	19	21	‐	‐

*Note*: “M” corresponds to the mean proportion, and ℙ corresponds to the proportion of scenarios with the percentage of optimal selections being above 40%, correct selection being above 60%, and overly toxic selection being below 25%. Results for the POCRM designs are based on 2000 simulations.

Overall, the proposed design under the escalation criterion for the allocation of patients (“next best”) has good accuracy properties—the average proportion of the optimal regimen selections is 39%, and the average proportion of acceptable selections is 68%. At the same time, the proportion of overly toxic selections is small—21%, on average—with proportion being below 25% in the half of all scenarios. Higher probabilities of making an overtoxic recommendations correspond to more toxic scenarios 4.3 to 8. At the same time, a particularly high proportion of overly toxic selections can be observed under scenarios 2.3 for which the highest dose of M is the optimal one but for 100 mg of N. This happens due to the selected prior probabilities of orderings—the orderings that are close to this scenarios (orderings 9 and 10 where the toxicity ordering goes throughout the grid) have low a‐priori probability, therefore, the considered sample size might not be always enough to overrule it. The same explains the low proportion of the optimal selections under this scenario. Hence, the likelihood of these scenarios should be considered when interpreting the results.

The average proportion of DLTs is below 33% under 18 out of 20 scenarios with the average proportion of DLTS being 28%. This corresponds to approximately middle of the target toxicity range, and is hence acceptable.

The allocation rule within the admissible set decreases the proportion of overly toxic selections for the same sample size due to a better exploration of the grid (eg, of regimens that might not be selected due to low probability of a‐priori ordering as we have seen above). Specifically, for the considered sample size, it allows to decrease the mean proportion of overly toxic selection from 21% to 18% and to increase the proportion of scenarios with overly toxic selection below 25% from 50% to 75%. Furthermore, the proportion of overly toxic selections under scenarios 2.3 drops from 33% to 19%. Importantly, the proportion of acceptable selections remains unchanged—68% with the price for not selecting the next best after each cohort being in the lower proportion of optimal selections from 39% to 36%. Similar patterns can be observed for other sample sizes (see the Supplementary Materials).

Comparing the proposed design to the 3+3 design, as expected, the 3+3 design results in noticeably lower proportion of optimal and correct regimens identifications. The probability to select the optimal combination is 15% lower, and the probability to select the correct combination is 21% lower compared to the proposed design under the “next best” allocation rule. The proportion of scenarios under which the optimal/correct selection is above 40%/60% drops to 15%/35% compared to 55% and 75%, respectively, under the “next best” allocation. Furthermore, the 3+3 design results, on average, in noticeably higher probability of recommending unsafe combination, 31% vs 21% for the POCRM, and 9% higher average proportion of patients experiencing a DLT. In 60% of scenarios this proportion is above 25%. This means that the 3+3 design results is less accurate and does not safeguard the patients to the same extent as the proposed design. However, it is important to mention that the 3+3 design results in noticeably lower expected number of patients than the proposed model‐based design due the early stopping rule—the average sample sizes ranges between 12 and 21. However, more toxic scenarios correspond to higher expected sample sizes. Furthermore, it worth reiterating that the maximum sample size for the 3+3 design is 54 patients, higher than for the proposed one, and this is the number of patients that a trial should be costed for were one to follow the 3+3 design.

Comparing the proposed design to the POCRM with model averaging, it is found that the model averaging approach tends to result in lower proportion of optimal selections (by 4%‐5%), similar proportion of correct selection (within 2%), and smaller proportion of overtoxic selections (6%‐7%). These conclusions are consistent across both allocation rules. This makes the POCRM with model averaging a viable alternative to the proposed design. Importantly, one could attempt to improve the operating characteristics of the model averaging approach as the parameters calibrated for the original proposal are used. However, this will be computationally expensive.

Overall, the proposed design was concluded to be able to identify the optimal and correct regimens with high probability. Given the trade‐off in the accuracy, safety, and costs, the decision to fix the sample sizes at n=48 was made given that the advantage of the six extra patients (ie, comparing to n=54, see the Supplementary Materials) were limited in terms of the accuracy.

## 7 DISCUSSION

Accurate identification of the optimal doses and schedules in early phase trials that balance safety and efficacy is of paramount importance for the whole drug development path. To find the optimal combinations of doses of several drugs and how these should be administered to the patients, various dosing and schedules parameters should be studied simultaneously as assuming one of them being fixed can result in suboptimal recommendations. However, this poses a number of challenges, both statistical and clinical. We have proposed an extension of the POCRM design and provided a step‐by‐step summary of how it was implemented in practice. The proposed design supports the decision‐making process during dose‐escalation and relies on a parametric model that allows borrowing of information across all regimens. As the result, it increases the likelihood of finding the dose with optimal benefit while safeguarding the patients by mitigating the risk of allocating patients to ineffective or overly toxic doses.

We have also proposed a novel approach to defining orderings and their prior probabilities to inform the POCRM design, utilizing the a‐priori clinical knowledge. This approach is more straightforward to communicate to the trial team than the alternative of specifying the probability of each complete ordering directly and ensures that the design reflects clinically plausible scenarios.

The proposed POCRM can incorporate prior clinical knowledge through the probability of each ordering. This approach is different to how the prior information is leveraged in the BLRM‐type approaches, which typically formally constructs a prior distribution on the model parameters themselves. At the same, being a model‐based approach too, the POCRM design can use an informative prior distribution on the model parameter. However, the one‐parameter working model used in the POCRM might not be flexible enough to achieve a good approximation of the prior toxicity estimates and sufficiently different level of uncertainty at various dose‐schedule combination. Moreover, under the proposed design of choosing the most likely ordering, this could be challenging as well, as changing the order will change how the prior information is included. The model‐average POCRM alternative might be a more natural way of achieving this if one would consider inclusion of in a similar to the BLRM‐type approaches way.

A number of tools, the DTP, probability grids, and complete output in several escalation paths that facilitate the communication of the properties of the designs have been developed. While simulations are an integral tool for understanding the statistical properties of the design, we stress that they should be considered together with the individual trial behavior and the investigation of the design should not be limited to simulations only. Giving more consideration to individual trial behavior is thought to resolve one of the concerns associated with model‐based designs, them being a “black box.”

Importantly, an implementation of such an advanced design in practice requires not only support by experienced statisticians but also a close collaboration with the whole trial team. For example, it took multiple iterations and meetings to agree on the plausible toxicity orderings, to define the corresponding prior probabilities, to agree on the scenarios for the simulations study, and define the operating characteristics that are of interest to the team. The developed supporting visualizations were the key in these meetings.

Finally, there is an increasing acceptance of more efficient statistical approaches in early phase clinical trials by pharmaceutical companies and academic institutions. The proposed design is currently being implemented in a clinical trial enrolment to which has commenced and has been reviewed by the FDA and MHRA. While implementing such a design does take resources, the use of more efficient methods earlier in the drug development path is expected to pay off later in development. Sharing such examples as presented in this work will contribute to improving the uptake of more efficient methods for advanced dose‐escalation trials.

## CONFLICT OF INTEREST

Thomas Goddemeier and Anja Victor are employees of Merck Healthcare KGaA, Darmstadt, Germany. Marianna Grinberg was an employee of Merck Healthcare KGaA, Darmstadt, Germany, at the time the reported work was conducted, and Marianna Grinberg is currently employee of UCB, Statistical Sciences and Innovation Division, Monheim, Germany. Marianna Grinberg and Anja Victor hold stocks of Merck Healthcare KGaA. Ioannis Gounaris is an employee of Merck Serono Ltd., Feltham, UK, an affiliate of Merck KGaA. Pavel Mozgunov and Thomas Jaki served as statistical consultants for Merck Healthcare KGaA.

## Supporting information


**Table S1** Operating characteristics of the proposed design under 3 sample sizes, n=42, 48, 54, and under two allocation rules
**Tables S2 to S4** Example of the output provided to the SRC after 0, 1, and 2 DLTs were observed in the first cohort of 3 patients on the starting regimen
**Figure S1** Example of the visualization of the probability of risk of toxicity for each combination‐schedule being in the underdosing, target, and overdosing interval used the grid representation of the regimens

## Data Availability

All simulations were conducted through the computing software JAGS and R.^47^ No new data have been used in this publication.
