# Supplementary material for: Practical implementation of the partial ordering continual reassessment method in a Phase I combination‐schedule dose‐finding trial
Source: Stat Med. 2022 Nov 25;41(30):5789–809. doi: 10.1002/sim.9594 (PMC10100035; doi:10.1002/sim.9594)
Supplement: Supplementary file 1 — Table S1 Operating characteristics of the proposed design under 3 sample sizes, n=42, 48, 54, and under two allocation rules Tables S2 to S4 Example of the output provided to the SRC after 0, 1, and 2 DLTs were observed in the first cohort of 3 patients on the starting regimen Figure S1 Example of the visualization of the probability of risk of toxicity for each combination‐schedule being in the underdosing, target, and overdosing interval used the grid representation of the regimens [file SIM-41-5789-s001.pdf]

Received: Added at production | Revised: Added at production | Accepted: Added at production

DOI: xxx/xxxx

ARTICLE TYPE

Supplementary Materials for Practical Implementation of the Partial Ordering Continual Reassessment Method in a Phase I Combination-Schedule Dose-Finding Trial

Pavel Mozgunov*[1] | Thomas Jaki[1,2] | Ioannis Gounaris[3] | Thomas Goddemeier[4] | Anja Victor[4] | Marianna Grinberg[4]

[1]MRC Biostatistics Unit, University of Cambridge, Cambridge, UK
[2]Department of Mathematics and Statistics, Lancaster University, Lancaster, UK
[3]Merck Serono Ltd., Feltham, UK, an affiliate of Merck KGaA, Feltham, UK
[4]Biostatistics, Epidemiology & Medical Writing, Merck Healthcare KGaA, Darmstadt, Germany

Correspondence
*Pavel Mozgunov, Email: pavel.mozgunov@mrc-bsu.cam.ac.uk

Present Address
MRC Biostatistics Unit, East Forvie Building, University of Cambridge, Cambridge CB2 0SR, United Kingdom.

Summary

KEYWORDS:
Combination Study, Dose-Finding, Dose-Schedule, Partial Ordering

SUPPLEMENTARY MATERIALS

Selected Orderings

1. 1, 2, 3, 6, 4, 5, 7, 8, 9, 10, 11, 12, 13, 16, 14, 15, 17, 18, 19, 20;
2. 1, 2, 3, 6, 7, 4, 5, 8, 9, 10, 11, 12, 13, 16, 17, 14, 15, 18, 19, 20;
3. 1, 2, 6, 3, 4, 7, 5, 8, 9, 10, 11, 12, 16, 13, 14, 17, 15, 18, 19, 20;
4. 1, 6, 2, 3, 7, 4, 5, 8, 9, 10, 11, 16, 12, 13, 17, 14 ,15 ,18, 19, 20
5. 1, 2, 6, 3, 7, 4, 8, 5, 9, 10, 11, 12, 16, 13, 17, 14, 18, 15, 19, 20;
6. 1, 2, 3, 6, 4, 7, 11, 12, 13, 16, 14, 17, 5, 8, 9, 10, 15, 18, 19, 20;
7. 1, 2, 6, 3, 7, 4, 11, 12, 16, 13, 17, 14, 5, 8, 9, 10, 15, 18, 19, 20;
8. 1, 2, 6, 3, 4, 7, 11, 12, 16, 13, 14, 17, 5, 8, 9, 10, 15, 18, 19, 20;
9. 1, 2, 6, 11, 3, 4, 7, 12, 13, 16, 5, 8, 14, 17, 9, 15, 18, 10, 19, 20;
10. 1, 2, 6, 11, 3, 16, 12, 13, 7, 4, 5, 8, 14, 17, 18, 15, 9, 10, 19, 20;

Prior Probabilities of Orderings

The a-priori probabilities of each of the ten orderings specified above that correspond to the pairwise probabilities reported in Table 3 are given by

$$(0.12, 0.16, 0.16, 0.09, 0.16, 0.07, 0.09, 0.07, 0.04, 0.04).$$

Operating Characteristics for $n = 42, 48, 54$

	1	2.1	2.2	2.3	3.1	3.2	3.3	4.1	4.2	4.3	4.4	4.5	5.1	5.2	5.3	5.4	6.1	6.2	7	8	M	ℙ
	Proposed POCRM Design – Next Best																					
	$n = 42$																					
Optimal	45	43	39	5	59	51	31	57	35	17	18	21	53	41	37	28	32	25	44	41	36	45
Correct	83	91	85	43	86	79	72	72	71	60	63	44	69	66	65	59	57	54	56	41	66	65
Overtoxic	0	4	8	32	11	13	18	19	25	32	30	34	25	29	15	28	39	35	40	31	23	50
% of DLTs	20	23	23	23	26	26	25	29	28	28	28	28	32	32	28	29	32	31	38	44	28	–
Early Stop	0	0	0	0	0	0	0	0	0	0	0	0	0	0	0	0	0	0	4	28	–	–
Av SS	42	42	42	42	42	42	42	42	42	42	42	42	42	42	42	42	42	42	41	37	–	–
	$n = 48$																					
Optimal	48	46	43	8	61	52	33	60	36	17	20	23	56	44	40	29	35	27	47	44	39	55
Correct	86	91	85	45	87	80	71	75	73	62	68	48	72	69	68	60	62	57	59	44	68	75
Overtoxic	0	4	8	33	10	13	19	18	24	32	27	31	23	26	13	27	35	32	37	27	21	50
% of DLTs	21	23	23	24	26	26	25	29	28	28	28	28	31	31	28	29	32	30	37	42	28	–
Early Stop	0	0	0	0	0	0	0	0	0	0	0	0	0	0	0	0	0	0	4	29	–	–
Av SS	48	48	48	48	48	48	48	48	48	48	48	48	48	48	48	48	48	48	47	41	–	–
	$n = 54$																					
Optimal	50	48	44	9	61	53	35	64	38	18	22	24	59	47	39	34	37	27	49	47	40	50
Correct	88	93	86	48	88	82	76	77	74	65	68	51	76	73	70	64	64	58	63	47	71	80
Overtoxic	0	4	7	31	10	12	16	16	23	30	27	29	19	23	13	23	32	30	34	23	19	65
% of DLTs	21	23	24	24	26	26	25	28	28	28	28	28	31	31	27	28	32	30	36	42	28	–
Early Stop	0	0	0	0	0	0	0	0	0	0	0	0	0	0	0	0	0	0	4	30	–	–
Av SS	54	54	54	54	54	54	54	54	54	54	54	54	54	54	54	54	54	54	53	45	–	–
	Proposed POCRM Design –Within Admissible Set																					
	$n = 42$																					
Optimal	41	35	30	11	50	46	32	53	37	14	22	19	55	38	28	28	32	18	44	43	34	35
Correct	81	88	80	52	84	78	74	72	74	64	66	46	72	67	61	64	62	55	59	43	67	75
Overtoxic	0	3	9	20	8	11	13	13	17	24	22	24	18	27	13	21	35	30	36	27	19	75
% of DLTs	20	23	23	24	26	25	24	28	27	27	27	28	32	31	27	28	33	31	39	45	28	–
Early Stop	0	0	0	0	0	0	0	0	0	0	0	0	0	0	0	0	0	0	4	30	–	–
Av SS	42	42	42	42	42	42	42	42	42	42	42	42	42	42	42	42	42	42	41	36	0–	–
	$n = 48$																					
Optimal	42	39	34	11	53	49	32	56	35	17	23	21	57	41	34	30	35	20	46	43	36	40
Correct	84	89	80	49	86	78	75	76	74	64	69	45	74	69	62	67	63	57	60	43	68	80
Overtoxic	0	2	9	19	8	10	12	12	19	24	21	28	18	25	14	20	35	29	36	27	18	75
% of DLTs	21	23	23	24	26	25	24	28	27	27	27	27	32	31	27	28	32	30	38	44	28	–
Early Stop	0	0	0	0	0	0	0	0	0	0	0	0	0	0	0	0	0	0	4	30	–	–
Av SS	48	48	48	48	48	48	48	48	48	48	48	48	48	48	48	48	48	48	47	41	–	–
	$n = 54$																					
Optimal	43	40	32	12	50	45	28	54	33	19	20	19	52	41	29	31	32	21	53	44	36	45
Correct	83	86	76	50	85	77	69	76	72	62	70	46	76	71	56	69	67	56	70	44	68	75
Overtoxic	0	3	8	18	6	6	13	8	14	19	16	21	15	18	13	15	26	23	25	22	14	95
% of DLTs	21	23	23	24	26	25	24	28	27	27	27	27	31	31	27	28	32	30	38	43	28	–
Early Stop	0	0	0	0	0	0	0	0	0	0	0	0	0	0	0	0	0	0	4	33	–	–
Av SS	54	54	54	54	54	54	54	54	54	54	54	54	54	54	54	54	54	54	52	44	–	–

TABLE S1 Operating characteristics for $n = 42, 48, 54$ following (i) the allocation according to the criterion (Next Best), (ii) the randomisation "Within Admissible Set". "M" corresponds to the mean proportion, and ℙ corresponds to the proportion of scenarios with the percentage of optimal selections being above 40%, correct selection being above 60%, and overly toxic selection being below 25%. Results are based on 2000 simulations.

	1	2.1	2.2	2.3	3.1	3.2	3.3	4.1	4.2	4.3	4.4	4.5	5.1	5.2	5.3	5.4	6.1	6.2	7	8	M	ℙ
	POCRM Design with Model Averaging – Next Best																					
	$n = 42$																					
Optimal	44	38	33	3	45	38	36	42	29	26	14	15	62	35	20	40	20	5	45	49	33	35
Correct	83	86	77	11	82	79	77	83	74	66	67	43	87	80	63	72	72	71	63	49	69	90
Overtoxic	0	3	8	23	11	14	13	9	16	18	23	28	9	12	16	15	22	19	33	23	15	90
% of DLTs	21	25	25	24	27	27	27	30	29	29	29	29	33	31	26	30	32	30	40	45	29	–
Early Stop	0	0	0	0	0	0	0	0	0	0	0	0	0	0	0	0	0	0	3	28	–	–
Av SS	42	42	42	42	42	42	42	42	42	42	42	42	42	42	42	42	42	42	41	37	–	–
	$n = 48$																					
Optimal	47	42	37	4	47	38	39	46	33	30	16	16	60	36	22	40	20	6	48	47	34	40
Correct	82	87	80	12	84	80	78	85	74	66	65	44	86	80	65	72	72	72	65	47	70	90
Overtoxic	0	3	7	26	9	13	12	8	15	17	23	31	9	12	16	16	22	20	31	22	15	90
% of DLTs	22	24	25	24	27	27	26	29	29	28	28	29	32	30	26	30	31	29	39	44	29	–
Early Stop	0	0	0	0	0	0	0	0	0	0	0	0	0	0	0	0	0	0	4	31	–	–
Av SS	48	48	48	48	48	48	48	48	48	48	48	48	48	48	48	48	48	48	47	41	–	–
	$n = 54$																					
Optimal	48	42	36	4	47	38	40	48	33	27	18	17	62	38	21	46	22	6	50	51	35	45
Correct	86	89	79	14	85	79	78	88	78	70	69	47	89	85	62	79	77	75	70	51	72	90
Overtoxic	0	3	10	24	9	14	13	6	13	15	20	28	7	10	17	11	18	18	26	17	14	90
% of DLTs	22	24	25	24	27	27	26	29	28	28	28	28	32	30	26	29	31	29	38	43	29	–
Early Stop	0	0	0	0	0	0	0	0	0	0	0	0	0	0	0	0	0	0	4	32	–	–
Av SS	54	54	54	54	54	54	54	54	54	54	54	54	54	54	54	54	54	54	53	45	–	–
	POCRM Design with Model Averaging – Within Admissible Set																					
	$n = 42$																					
Optimal	32	28	25	5	34	34	27	42	36	22	20	18	60	37	23	37	26	13	44	47	31	20
Correct	70	78	75	17	82	77	66	80	75	54	66	32	84	75	62	66	70	67	65	47	65	85
Overtoxic	0	3	6	17	6	6	6	7	8	10	17	15	9	16	7	16	24	17	31	22	12	95
% of DLTs	18	20	20	20	22	22	21	25	24	24	23	25	29	27	26	26	29	27	36	43	25	–
Early Stop	0	0	0	0	0	0	0	0	0	0	0	0	0	0	0	0	0	0	4	31	–	–
Av SS	42	42	42	42	42	42	42	42	42	42	42	42	42	42	42	42	42	42	41	36	–	–
	$n = 48$																					
Optimal	33	30	28	5	39	37	30	44	37	25	22	17	57	42	24	39	23	15	44	46	32	25
Correct	72	80	77	16	82	80	67	83	75	61	66	31	83	79	61	67	66	66	64	46	66	90
Overtoxic	0	2	6	18	5	6	6	5	9	7	17	15	11	13	8	14	25	17	31	21	11	90
% of DLTs	18	20	20	20	22	22	21	24	24	23	22	24	28	27	25	26	28	26	35	42	25	–
Early Stop	0	0	0	0	0	0	0	0	0	0	0	0	0	0	0	0	0	0	5	34	–	–
Av SS	48	48	48	48	48	48	48	48	48	48	48	48	48	48	48	48	48	48	46	40	–	–
	$n = 54$																					
Optimal	35	31	26	5	39	34	31	48	39	23	26	21	60	42	32	44	27	17	46	51	34	30
Correct	77	85	80	17	83	79	70	85	81	61	72	34	86	82	67	72	70	74	67	51	70	90
Overtoxic	0	2	5	21	6	7	7	4	6	9	14	11	9	13	7	13	24	12	29	16	10	95
% of DLTs	18	20	20	19	22	22	21	24	24	23	22	24	28	27	25	26	28	26	35	41	25	–
Early Stop	0	0	0	0	0	0	0	0	0	0	0	0	0	0	0	0	0	0	4	34	–	–
Av SS	54	54	54	54	54	54	54	54	54	54	54	54	54	54	54	54	54	54	53	44	–	–

TABLE S2 Operating characteristics of the POCRM design with model averaging for $n = 42, 48, 54$ following (i) the allocation according to the criterion (Next Best), (ii) the randomisation "Within Admissible Set". "M" corresponds to the mean proportion, and $\mathbb{P}$ corresponds to the proportion of scenarios with the percentage of optimal selections being above 40%, correct selection being above 60%, and overly toxic selection being below 25%. Results are based on 2000 simulations.

Example of model recommendation output

As mentioned in the main body of the manuscript, at the planning stage of the trial, it is crucial to ensure that the proposed model leads to intuitive escalation/de-escalation decision throughout the trial. One way to ensure this is through the decision-tree presented in the main manuscript. In addition, we also provided the complete output of the model that will be shared with the trial team during the conduct of the trial. This provides a more detailed look at why the proposed model recommends the regimens as per the decision-tree but also to ensure that the output is relevant to the team to ensure efficient decision-making.

There are two types of output that were provided to the team: (i) the complete table of the estimated characteristics for all twenty regimens, and (ii) a figure with the probabilities of each regimen being overtoxic, undertoxic, and in the target toxicity range.

The detailed table were agreed to include the following information for each regimen:

- Number of patients and DLTs;
- Mean estimated toxicity risk;
- 95% Credible interval
- Probability of overdosing: $\mathbb{P}\left(p_{ir^\star} > 0.35\right)$
- Probability being in the target range: $\mathbb{P}\left(0.20 \leq p_{ir^\star} \leq 0.35\right)$

We have also used the following colour-coding to aid the communication of the output table:
The following colour-coding is used

- If the overdose probability is above 25%, the corresponding cell is red.
- If the overdose probability is below 25%, there are two possibilities, the cell can be green or blue with the convention that all of these are safe according to the model but green are preferred for the escalation as follows:
  - Safe combinations corresponding to more than doubling of the total average and single dose are blue.
  - If the combination is escalated [within the chosen most likely ordering], the escalated combinations are green.
  - If the combinations is de-escalated and there are more than six safe combinations, the combinations that correspond to less than halving of the total average dose of M or 200mg dose of N are preferred (and hence are in green).

Additionally, we have provided a figure displaying for each regimen being overtoxic(red, $\mathbb{P}\left(p_{ir^\star} > 0.35\right)$) , being in the target range (green, $\mathbb{P}\left(0.20 \leq p_{ir^\star} \leq 0.35\right)$), and being undertoxic (blue, $\mathbb{P}\left(p_{ir^\star} \leq 0.20\right)$. This figure uses the same combination-schedule grid given in Table 2 of the main body of the manuscript. An example of such figure after 1 DLT was observed for the first cohort of 3 patients at the starting combination (18) is given in Figure S1. The threshold of 75% is provided to illustrated which of the regimens are deemed overly toxic (using the probability threshold of 25%) and cannot be selected.

0 DLTs at (18)

	200N, 30M, S1<br>n= 0 | DLTs= 0<br>Mean Tox= 0<br>95%CI=(0,0.26)<br>Overdose= 1%<br>Target= 3.3%			200N, 60M, S1<br>n= 0 | DLTs= 0<br>Mean Tox= 0.01<br>95%CI=(0,0.35)<br>Overdose= 2.4%<br>Target= 6.1%		200N, 90M, S1<br>n= 3 | DLTs= 0<br>Mean Tox= 0.03<br>95%CI=(0,0.44)<br>Overdose= 5.5%<br>Target= 10.6%		200N, 130M, S1<br>n= 0 | DLTs= 0<br>Mean Tox= 0.1<br>95%CI=(0,0.58)<br>Overdose= 15.2%<br>Target= 18.4%	200N, 180M, S1<br>n= 0 | DLTs= 0<br>Mean Tox= 0.17<br>95%CI=(0,0.66)<br>Overdose= 25.3%<br>Target= 19.3%
200N, 30M, S2<br>n= 0 | DLTs= 0<br>Mean Tox= 0<br>95%CI=(0,0.18)<br>Overdose= 0.4%<br>Target= 1.7%		200N, 60M, S2<br>n= 0 | DLTs= 0<br>Mean Tox= 0<br>95%CI=(0,0.22)<br>Overdose= 0.7%<br>Target= 2.4%	200N, 90M, S2<br>n= 0 | DLTs= 0<br>Mean Tox= 0.01<br>95%CI=(0,0.3)<br>Overdose= 1.6%<br>Target= 4.5%		200N, 130M, S2<br>n= 0 | DLTs= 0<br>Mean Tox= 0.02<br>95%CI=(0,0.39)<br>Overdose= 3.6%<br>Target= 8.1%		200N, 180M, S2<br>n= 0 | DLTs= 0<br>Mean Tox= 0.05<br>95%CI=(0,0.5)<br>Overdose= 8.2%<br>Target= 0%		
	100N, 60M, S1<br>n= 0 | DLTs= 0<br>Mean Tox= 0<br>95%CI=(0,0.01)<br>Overdose= 0%<br>Target= 0%			100N, 60M, S1<br>n= 0 | DLTs= 0<br>Mean Tox= 0<br>95%CI=(0,0.03)<br>Overdose= 0%<br>Target= 0.1%		100N, 90M, S1<br>n= 0 | DLTs= 0<br>Mean Tox= 0<br>95%CI=(0,0.07)<br>Overdose= 0.1%<br>Target= 0.4%		100N, 130M, S1<br>n= 0 | DLTs= 0<br>Mean Tox= 0<br>95%CI=(0,0.12)<br>Overdose= 0.2%<br>Target= 0.8%	100N, 180M, S1<br>n= 0 | DLTs= 0<br>Mean Tox= 0<br>95%CI=(0,0.15)<br>Overdose= 0.3%<br>Target= 1.2%
100N, 30M, S2<br>n= 0 | DLTs= 0<br>Mean Tox= 0<br>95%CI=(0,0)<br>Overdose= 0%<br>Target= 0%		100N, 60M, S2<br>n= 0 | DLTs= 0<br>Mean Tox= 0<br>95%CI=(0,0)<br>Overdose= 0%<br>Target= 0%	100N, 90M, S2<br>n= 0 | DLTs= 0<br>Mean Tox= 0<br>95%CI=(0,0.02)<br>Overdose= 0%<br>Target= 0.1%		100N, 130M, S2<br>n= 0 | DLTs= 0<br>Mean Tox= 0<br>95%CI=(0,0.05)<br>Overdose= 0%<br>Target= 0.2%		100N, 180M, S2<br>n= 0 | DLTs= 0<br>Mean Tox= 0<br>95%CI=(0,0.1)<br>Overdose= 0.1%<br>Target= 0.5%		

TABLE S3 Example of the output provided to the SRC if the first cohort of 3 patients experienced no DLTs

1 DLT at (18)

	200N, 30M, S1<br>n= 0 | DLTs= 0<br>Mean Tox= 0.14<br>95%CI=(0,0.56)<br>Overdose= 13.8%<br>Target= 20.4%			200N, 60M, S1<br>n= 0 | DLTs= 0<br>Mean Tox= 0.17<br>95%CI=(0.01,0.6)<br>Overdose= 18.4%<br>Target= 23.5%		200N, 90M, S1<br>n= 3 | DLTs= 1<br>Mean Tox= 0.31<br>95%CI=(0.03,0.71)<br>Overdose= 41.2%<br>Target= 26.9%		200N, 130M, S1<br>n= 0 | DLTs= 0<br>Mean Tox= 0.41<br>95%CI=(0.07,0.77)<br>Overdose= 58.5%<br>Target= 25.5%	200N, 180M, S1<br>n= 0 | DLTs= 0<br>Mean Tox= 0.5<br>95%CI=(0.13,0.81)<br>Overdose= 74.9%<br>Target= 20.1%
200N, 30M, S2<br>n= 0 | DLTs= 0<br>Mean Tox= 0.06<br>95%CI=(0,0.44)<br>Overdose= 5.5%<br>Target= 11.6%		200N, 60M, S2<br>n= 0 | DLTs= 0<br>Mean Tox= 0.08<br>95%CI=(0,0.48)<br>Overdose= 7.5%<br>Target= 14.3%	200N, 90M, S2<br>n= 0 | DLTs= 0<br>Mean Tox= 0.11<br>95%CI=(0,0.52)<br>Overdose= 10.2%<br>Target= 17.3%		200N, 130M, S2<br>n= 0 | DLTs= 0<br>Mean Tox= 0.21<br>95%CI=(0.01,0.63)<br>Overdose= 24.4%<br>Target= 26.3%		200N, 180M, S2<br>n= 0 | DLTs= 0<br>Mean Tox= 0.26<br>95%CI=(0.02,0.67)<br>Overdose= 31.9%<br>Target= 28.2%		
	100N, 60M, S1<br>n= 0 | DLTs= 0<br>Mean Tox= 0<br>95%CI=(0,0.15)<br>Overdose= 0.3%<br>Target= 1.2%			100N, 60M, S1<br>n= 0 | DLTs= 0<br>Mean Tox= 0<br>95%CI=(0,0.19)<br>Overdose= 0.5%<br>Target= 1.9%		100N, 90M, S1<br>n= 0 | DLTs= 0<br>Mean Tox= 0.02<br>95%CI=(0,0.32)<br>Overdose= 1.9%<br>Target= 5.4%		100N, 130M, S1<br>n= 0 | DLTs= 0<br>Mean Tox= 0.03<br>95%CI=(0,0.36)<br>Overdose= 2.8%<br>Target= 7.1%	100N, 180M, S1<br>n= 0 | DLTs= 0<br>Mean Tox= 0.04<br>95%CI=(0,0.4)<br>Overdose= 3.9%<br>Target= 9.2%
100N, 30M, S2<br>n= 0 | DLTs= 0<br>Mean Tox= 0<br>95%CI=(0,0.01)<br>Overdose= 0%<br>Target= 0%		100N, 60M, S2<br>n= 0 | DLTs= 0<br>Mean Tox= 0<br>95%CI=(0,0.06)<br>Overdose= 0.1%<br>Target= 0.3%	100N, 90M, S2<br>n= 0 | DLTs= 0<br>Mean Tox= 0<br>95%CI=(0,0.11)<br>Overdose= 0.1%<br>Target= 0.7%		100N, 130M, S2<br>n= 0 | DLTs= 0<br>Mean Tox= 0<br>95%CI=(0,0.24)<br>Overdose= 0.8%<br>Target= 2.8%		100N, 180M, S2<br>n= 0 | DLTs= 0<br>Mean Tox= 0<br>95%CI=(0,0.28)<br>Overdose= 1.3%<br>Target= 4%		

TABLE S4 Example of the output provided to the SRC if the first cohort of 3 patients experienced 1 DLT

2 DLT at (18)

<table>
<tr><td></td><td>200N, 30M, S1
n= 0 | DLTs= 0
Mean Tox= 0.32
95%CI=(0.03,0.73)
Overdose= 42.4%
Target= 27.7%</td><td></td><td></td><td>200N, 60M, S1
n= 0 | DLTs= 0
Mean Tox= 0.36
95%CI=(0.05,0.75)
Overdose= 50.3%
Target= 26.8%</td><td></td><td>200N, 90M, S1
n= 3 | DLTs= 2
Mean Tox= 0.51
95%CI=(0.14,0.83)
Overdose= 76.5%
Target= 22.1%</td><td></td><td>200N, 130M, S1
n= 0 | DLTs= 0
Mean Tox= 0.6
95%CI=(0.21,0.87)
Overdose= 88.3%
Target= 9.7%</td><td>200N, 180M, S1
n= 0 | DLTs= 0
Mean Tox= 0.67
95%CI=(0.3,0.89)
Overdose= 95.4%
Target= 5.2%</td></tr>
<tr><td>200N, 30M, S2
n= 0 | DLTs= 0
Mean Tox= 0.2
95%CI=(0.01,0.64)
Overdose= 23.5%
Target= 24.5%</td><td></td><td>200N, 60M, S2
n= 0 | DLTs= 0
Mean Tox= 0.24
95%CI=(0.01,0.67)
Overdose= 29%
Target= 26.3%</td><td>200N, 90M, S2
n= 0 | DLTs= 0
Mean Tox= 0.28
95%CI=(0.02,0.7)
Overdose= 35.3%
Target= 27.4%</td><td></td><td>200N, 130M, S2
n= 0 | DLTs= 0
Mean Tox= 0.41
95%CI=(0.07,0.78)
Overdose= 58.8%
Target= 24.8%</td><td></td><td>200N, 180M, S2
n= 0 | DLTs= 0
Mean Tox= 0.46
95%CI=(0.1,0.8)
Overdose= 67.6%
Target= 21.6%</td><td></td><td></td></tr>
<tr><td></td><td>100N, 60M, S1
n= 0 | DLTs= 0
Mean Tox= 0.03
95%CI=(0,0.36)
Overdose= 2.8%
Target= 6.6%</td><td></td><td></td><td>100N, 60M, S1
n= 0 | DLTs= 0
Mean Tox= 0.04
95%CI=(0,0.41)
Overdose= 4.3%
Target= 9.1%</td><td></td><td>100N, 90M, S1
n= 0 | DLTs= 0
Mean Tox= 0.11
95%CI=(0,0.54)
Overdose= 11.4%
Target= 17.1%</td><td></td><td>100N, 130M, S1
n= 0 | DLTs= 0
Mean Tox= 0.14
95%CI=(0,0.57)
Overdose= 14.8%
Target= 19.8%</td><td>100N, 180M, S1
n= 0 | DLTs= 0
Mean Tox= 0.17
95%CI=(0,0.61)
Overdose= 18.8%
Target= 22.3%</td></tr>
<tr><td>100N, 30M, S2
n= 0 | DLTs= 0
Mean Tox= 0
95%CI=(0,0.1)
Overdose= 0.2%
Target= 0%</td><td></td><td>100N, 60M, S2
n= 0 | DLTs= 0
Mean Tox= 0
95%CI=(0,0.22)
Overdose= 0.8%
Target= 2.5%</td><td>100N, 90M, S2
n= 0 | DLTs= 0
Mean Tox= 0.01
95%CI=(0,0.3)
Overdose= 1.7%
Target= 4.4%</td><td></td><td>100N, 130M, S2
n= 0 | DLTs= 0
Mean Tox= 0.01
95%CI=(0,0.46)
Overdose= 6.2%
Target= 11.6%</td><td></td><td>100N, 180M, S2
n= 0 | DLTs= 0
Mean Tox= 0.01
95%CI=(0,0.5)
Overdose= 8.5%
Target= 14.3%</td><td></td><td></td></tr>
</table>

TABLE S5 Example of the output provided to the SRC if the first cohort of 3 patients experienced 2 DLTs

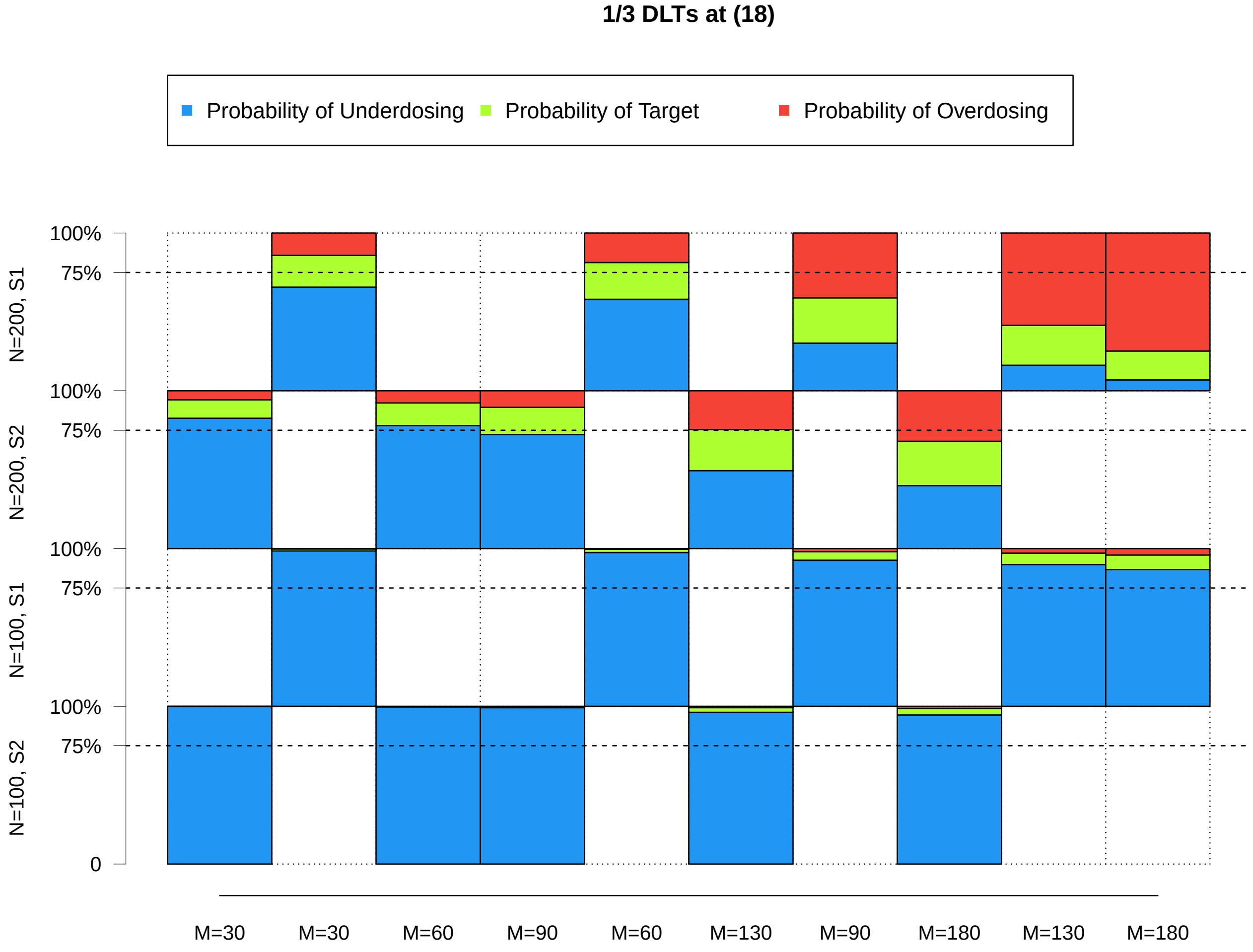

FIGURE S1 Example of the visualisation of the probability of risk of toxicity for each combination-schedule being in the underdosing, target, and overdosing interval used the grid representation of the regimens. The example is displayed after 1 DLT was observed for the first cohort of 3 patients at the starting combination (18).

□
